# Parasites and diet of *Serrasalmus maculatus* in a hydroelectric reservoir in Brazil

**DOI:** 10.1590/S1984-29612022013

**Published:** 2022-03-23

**Authors:** Bianca da Silva Miguel, Lidiane Franceschini, Letícia de Oliveira Manoel, Bruna Caroline Kotz Kliemann, Rosilene Luciana Delariva, Igor Paiva Ramos

**Affiliations:** 1 Programa de Pós-graduação em Ciências Biológicas (Zoologia), Instituto de Biociências, Universidade Estadual Paulista – UNESP, Botucatu, SP, Brasil; 2 Laboratório de Ecologia de Peixes, Departamento de Biologia e Zootecnia, Faculdade de Engenharia de Ilha Solteira, Universidade Estadual Paulista – UNESP, Ilha Solteira, SP, Brasil; 3 Centro de Ciências Biológicas e da Saúde, Universidade Estadual do Oeste do Paraná – UNIOESTE, Cascavel, PR, Brasil

**Keywords:** Ectoparasites, Anacanthorus, Mymarothecium, freshwater fish, piranha, Ectoparasitos, Anacanthorus, Mymarothecium, peixe de água doce, piranha

## Abstract

*Serrasalmus maculatus* is a species of piranha which, despite being abundant in a reservoir environment, has few studies related to its parasitological and diet aspects. Thus, we aimed to document the parasitic fauna and diet of the *S. maculatus* in a hydroelectric reservoir in Brazil. In addition, we perform two literature reviews for the Neotropical region, recording the parasitic fauna already associated with *S. maculatus* and the occurrence of parasite genera identified in this study parasitizing Characiformes from other aquatic systems. Thirty-one hosts were collected with gillnets, from August 2014 to September 2016. *Serrasalmus maculatus* had a piscivorous feeding habit and a low richness parasitic component community, including two taxa of monogeneans, *Anacanthorus lepyrophallus* and *Mymarothecium* sp.; no endohelminths were observed. Data from the literature review, together with the findings of the study, showed that *S. maculatus* in the Neotropical region harbors 25 helminth taxa, with the monogenean being the most prevalent parasitic group and Brazil is the country with the most reports of the parasitic genera. These findings provide information on the relationships between diet, social behavior, and parasitic fauna of *S. maculatus* and on the patterns of distribution and infection of the observed parasite rates.

## Resumo

*Serrasalmus maculatus* é uma espécie de piranha que, a despeito de ser abundante em ambiente de reservatório, possui poucas informações sobre seus aspectos parasitológicos e dieta. Assim, o presente estudo objetivou documentar a fauna parasitária e a dieta de *S. maculatus* em um reservatório brasileiro. Além disso, foram realizadas duas revisões literárias para a região Neotropical, registrando a fauna parasitária, já associada a *S. maculatus* e a ocorrência dos gêneros parasitários identificados neste estudo, registrados em outros peixes Characiformes em outros sistemas aquáticos. Foram coletados 31 hospedeiros com redes de espera entre agosto de 2014 e setembro de 2016. *Serrasalmus maculatus* apresentou hábito alimentar piscívoro e comunidade componente parasitária com baixa riqueza, incluindo dois táxons de monogenéticos, *Anacanthorus lepyrophallus* e *Mymarothecium* sp.; não foram observados endohelmintos. Dados da revisão da literatura, juntamente com os achados deste estudo, mostraram que *S. maculatus*, na região Neotropical, abriga 25 táxons de helmintos, sendo monogenéticos o grupo de parasitos mais prevalente, e o Brasil o país com mais relatos de parasitos. Estes resultados fornecem informações sobre as relações entre dieta, o comportamento social e a fauna parasitária de *S. maculatus* e sobre os padrões de distribuição e infecção das taxas de parasitos observadas.

## Introduction

Parasites can influence local communities by affecting host physiology, morphology, reproduction, and behaviour, thereby affecting population, community, and ecosystem structures, and host behaviours (e.g., feeding habits and predator-prey relationships) in turn, can affect the structures of parasite communities (Timi & Poulin, 2020). However, even though the ecological relevance of parasitism is widely recognised, many studies have neglected the effects of these organisms on their hosts (Timi & Poulin, 2020). For example, even though Brazil harbours a megadiverse freshwater ichthyofauna (~3500 species) (Froese & Pauly, 2020a), the parasitology of only 13% of the region’s species has been evaluated, of which the majority are economically important species. Nevertheles little is known about the parasitology of fish species with low commercial importance (Eiras et al., 2010, 2011).

The piranha, or pirambeba, *Serrasalmus maculatus* (Kner, 1858) is a medium-sized freshwater fish belonging to Characiformes, that is widely distributed in South America, throughout both the Amazon and Paraguay-Paraná River basins (Froese & Pauly, 2020b). The species is piscivorous, preferentially consuming fish musculature, fins, and scales. Eventually, invertebrates are the speciesmost common prey (Agostinho & Marques, 2001; Agostinho et al., 2003; Villares et al., 2008). It is also generally gregarious and, although has low economic importance, is one of the most abundant species in hydroelectric reservoirs, because readily adapts to artificial lentic environments (Sazima & Machado, 1990; Hoffmann et al., 2005; Behr & Signor, 2008). Despite the abundance of *S. maculatus* in hydroelectric reservoirs, there are few studies on its parasitological aspects.

Most studies of the parasitology of *S. maculatus* have focused on populations in the Upper Paraná River floodplain region (Pavanelli et al., 1997, 2004; Takemoto et al., 2009; Casali & Takemoto, 2016; Moreira et al., 2019), and few studies have examined this species ecology or parasitology in artificial environments. In addition, considering the diet is an important factor in host-parasite interactions and hosts with more diverse diets tend to be more susceptible to endoparasite infections (Lima et al., 2016), we aimed (i) document the parasitic fauna and (ii) characterize the diet of *S. maculatus* in a hydroelectric reservoir in Brazil. We targeted also (iii) to verify the parasite fauna already associated with *S. maculatus* in the Neotropical region; and (iv) the occurrence of parasite genera - identified in the present study - in characiform fishes from other aquatic systems (natural or artificial) in the Neotropical region.

## Material and Methods

### Study area

**Figure 1:**
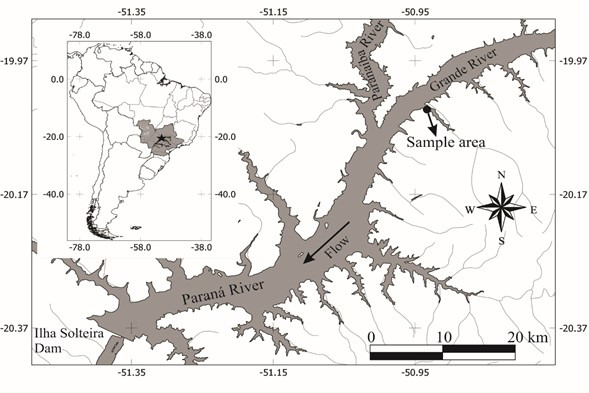
Study area on Ilha Solteira hydroelectric reservoir, Upper Paraná River basin, São Paulo state, Brazil (Campos et al., 2020).

The Ilha Solteira hydroelectric reservoir is an accumulation basin that was formed in 1978 and is situated along the Upper Paraná River, between the states of São Paulo, Minas Gerais, and Mato Grosso do Sul, Brazil (Figure 1). With a mean depth of 17.6 m, maximum volume of21.06 × 10^9^ m^3^, hydrographic basin area of 1195 km^2^, and residence time of 46.7 days, it is one of the largest artificial reservoirs in the neotropics (Garcia et al., 2014). For the present study, host sampling was conducted in the Can-Can arm in municipality of Santa Clara D’Oeste, São Paulo state, Brazil (50° 55ʹ 59.65″ W and 20° 02ʹ 30.54″ S).

### Host sampling

*Serrasalmus maculatus* specimens were collected using gill nets (3, 4, 5, 6, 7, 8, 10, 12 and 14 cm between non-adjacent nodes) between August 2014 to September 2016 (authorization SISBio nº 42229-1). The collected specimens were euthanized (Authorization CEUA/FEIS nº 001/2014 and Certified SisGen A9038DB) and identified as described by Ota et al. (2018). The total weight (g, with viscera) and standard length (cm, from snout to last vertebra) of each specimen were recorded, and the fish were subsequently individually stored in plastic bags, frozen and sent to the laboratory for additional analyses. All measurements are expressed as the mean ± standard deviation followed by the range.

### Parasitological procedures

The organs (skin, fins, nasal cavities, gills, eyes, heart, liver, gonads, intestines, swim bladder, spleen, gallbladder, and mesentery) were analysed for parasitological procedures, using a stereomicroscope, and parasites preserved in 70% ethanol or mounted on semipermanent slides using Gray and Wess medium. The parasite specimens were then subject to morphological analysis, using a computerised image analysis system with differential interference contrast (DIC) - LAS V3 (Leica Application Suite V3; Leica Microsystems, Wetzlar, Germany) and identified according to Kritsky et al. (1992) and Kritsky et al. (1996). Parasite prevalence (P, in percentage), mean intensity of infestation (MII), and mean abundance (MA) were then calculated according to Bush et al. (1997). Mean intensity of infestation and mean abundance are expressed as the mean ± standard error followed by the range.

The host and parasite voucher specimens were deposited in the Fish Collection of São Paulo State University (UNESP), Campus of São José do Rio Preto, São Paulo state, Brazil (DZSJRP 21374), and the Helminthological Collection of the Institute of Bioscience, Section of Parasitology, UNESP, Campus of Botucatu, São Paulo state, Brazil, (*Mymarothecium* sp. - CHIBB 652 L‒655 L; *Anacanthorus lepyrophallus* - CHIBB 656 L‒663 L), respectively.

### Literature review

Two literature reviews were conducted to verify the parasite fauna already associated with *S. maculatus* in the Neotropical region; and to verify the occurrence of parasite genera - identified in the present study - in characiform fishes from other aquatic systems (natural or artificial) in the Neotropical region. In the first review, we collected data on the helminth fauna previously reported for *S. maculatus* and its synonymy (= *Serrasalmus spilopleura* Kner, 1860) from the Neotropical region, from the first report in 1997 to 2021. In the second review, we collected data regarding the occurrence of monogenean species belonging to *Anacanthorus* and *Mymarothecium* genera in *S. maculatus*, as well as in other characiforms from the Neotropical region, from the first report of each genus (1965 to 2021 for *Anacanthorus*, and 1996 to 2021 for *Mymarothecium*).

The literature reviews were performed by searching relevant databases (SciELO, ISI, Scopus, Google Scholar, and WoRMS) for relevant terms: *Serrasalmus*, piranha, pirambeba, fish parasite, helminth, Monogenea, Dactylogyridae, Gyrodactylidae, Nematoda, Cestoda, Acanthocephala, Trematoda, Digenea, digenetic, digenean, monogenetic, monogenean, cestode, acanthocephalan, *Anacanthorus*, and *Mymarothecium*. All common names were searched using both singular and plural forms in English, Portuguese, and Spanish.

### Diet analysis

The stomachs of the host specimens were removed, fixed in 4% formaldehyde, and preserved in 70% alcohol, and stomach contents were analysed using an optical stereomicroscope. Recovered food items were quantified using the volumetric method (displacement of each measured food item from stomach contents using a gridded Petri dish) (Hyslop, 1980). Glass slides were used to compress food items to 1.0 mm in height, and the number of quadrants occupied by each food item was multiplied by 0.001 to calculate the volume in ml (Hellawell & Abel, 1971). All food items were identified to lowest possible taxonomic (Bicudo & Bicudo, 1970; Mugnai et al., 2010; Ota et al., 2018).

## Results

The weight and standard length of the 31 *S. maculatus* specimens ranged from 32.24 to 650.40 g (139.95 ± 24.42 g) and from 9.5 to 24.0 cm (14.44 ± 0.53), respectively.

The richness of the *S. maculatus* component parasite community was low and included two monogenean ectoparasites from gills, belonging to Dactylogyridae: *Anacanthorus lepyrophallus* (P = 84.2%, MII = 7.51 ± 1.50 [1–35], MA = 6.54 ± 1.38 [0–35]) and *Mymarothecium* sp. (P = 10.5%, MII = 2.33 ± 1.33 [1–7], MA = 0.22 ± 0.92 [0–7]). A total of 210 specimens were collected, and the overall P, MII, and MA of the parasites were 87.09%, 7.78 ± 1.48 (1–35), and 6.77 ± 1.37 (0–35), respectively. No endohelminths were recorded.

**Figure 2:**
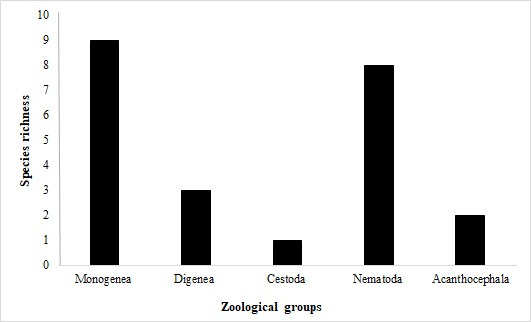
Taxonomic distribution of parasitic fauna reported from the piranha *Serrasalmus maculatus* in Neotropical region.

**Figure 3:**
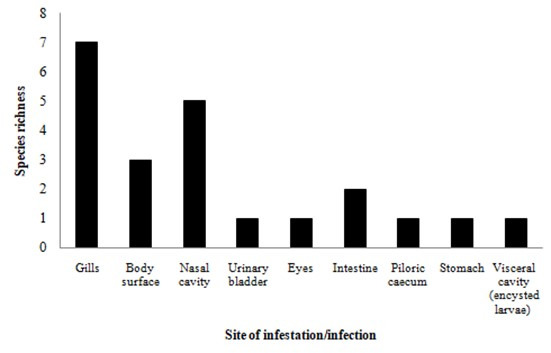
Species richness of parasites reported in *Serrasalmus maculatus* from Neotropical region, according with their site of infection.

Data from the literature review jointly with data from the specimens evaluated here demonstrated that *S. maculatus* in the Neotropical region harbour 25 helminth taxa (Table 1). Of these 25 taxa, 10 are monogeneans, nine nematodes, three digeneans, two acanthocephalans, and one cestode (Figure 2). Monogeneans most commonly infect host gills, followed by the nasal cavities and body surface (mucus), whereas the endohelminth groups with higher richness, nematodes and acanthocephalans, most commonly infect host intestines (Table 1 and Figure 3). Furthermore, the majority (16/25) of parasite taxa were reported from the Upper Paraná River floodplain in Brazil.

**Table 1:** Helminth parasites reported from the piranha *Serrasalmus maculatus* * in Neotropical region.

Parasites	Locality	Site of infection	Reference
Platyhelminthes			
Monogenea			
*Anacanthorus lepyrophallus* (Kritsky, Boeger & Van Every,1992)	Upper Paraná River floodplain, Paraná state, Brazil; Ilha Solteira reservoir, Grande River, Upper Paraná River basin, São Paulo state, Brazil	Gills	Moreira et al. (2019), **Present study**
*Anacanthorus paraxaniophallus* (Moreira, Carneiro, Ruz & Luque, 2019)	Miranda River, Pantanal, Mato Grosso do Sul state	Gills	Moreira et al. (2019)
*Anacanthorus sciponophallus* (Van Every & Kritsky, 1992)	Batalha River and Peixe River, Upper Paraná River basin, São Paulo state, Brazil	Body surface, gills, and nasal cavity	Dias et al. (2017)
*Kritskyia annakohnae* (Boeger, Tanaka & Pavanelli, 2001)	Baía River, Upper Paraná River basin, Brazil; Upper Paraná River	Urinary bladder and ureters; Unspecified	Boeger et al. (2001), Pavanelli et al. (2004), Takemoto et al. (2009), Casali & Takemoto (2016)
	floodplain in Mato Grosso do Sul state (next to municipality of Porto Rico), Paraná state, Brazil		
*Mymarothecium* sp.	Ilha Solteira reservoir, Grande River, Upper Paraná River basin, São Paulo state, Brazil	Gills	**Present study**
*Notothecium deleastoideum* (Kritsky, Boeger & Jégu, 1998	Peixe River, Upper Paraná River basin, São Paulo state, Brazil	Gills and body surface	Dias et al. (2017)
*Notozothecium minus* Boeger & Kritsky, 1988(=*Notozothecium minor* Boeger & Kritsky, 1988)	Batalha River, Upper Paraná River basin, São Paulo state, Brazil	Body surface, gills and nasal cavity	Dias et al. (2017)
*Rhinoxenus euryxenus* (Domingues & Boeger, 2005)	Paraná River, Paraná state, Brazil; Colastiné River, Sauce Viejo, Santa Fe Province, Argentina; Paraná Viejo River, Sauce Viejo, Santa Fe Province, Argentina	Nasal cavity	Domingues & Boeger (2005), Rossin et al. (2019)
*Rhinoxenus paranaensis* (Rossin & Timi, 2019)	Paraná River, Entre Ríos Province, Argentina; La Chancha Lagoon, Sauce Viejo, Santa Fe Province, Argentina; Lima, Partido de Zárate, Buenos Aires Province, Argentina	Nasal cavity	Rossin et al. (2019)
*Rhinoxenus piranhus* (Kritsky, Boeger & Thatcher, 1988)	Paraná River, Paraná state, Brazil; Batalha River, Upper Paraná River basin, São Paulo state, Brazil	Nasal cavity; body surface, gills, and nasal cavity	Domingues & Boeger (2005), Dias et al., (2017), Rossin et al. (2019)
Trematoda, Digenea			
Digenea gen. sp.	Upper Paraná River floodplain, Paraná state, Brazil	Unspecified	Pavanelli et al. (1997)
*Austrodiplostomum compactum* (Lutz, 1928) Dubois, 1970	Rosana reservoir, Paranapanema River, Brazil	Eyes	Yamada et al. (2008)
*Prosorhynchus piranhus* (Thatcher, 1999)	Upper Paraná River floodplain, Paraná state, Brazil	Unspecified	Pavanelli et al. (2004), Takemoto et al. (2009)
Cestoda			
*Proteocephalus serrasalmus* (Rego & Pavanelli, 1990)	Upper Paraná River floodplain, Paraná state, Brazil	Unspecified	Pavanelli et al. (1997), Pavanelli et al. (2004), Takemoto et al. (2009)
Acanthocephala			
Acanthocephala gen. sp.	Upper Paraná River floodplain, Paraná state, Brazil	Unspecified	Pavanelli et al. (1997), Pavanelli et al. (2004), Takemoto et al. (2009)
*Echinorhynchus* sp.	Upper Paraná River	Intestine and stomach	Casali & Takemoto (2016)
	floodplain in Mato Grosso do Sul state (next to municipality of Porto Rico), Paraná state, Brazil		
Nematoda			
Capillariidae gen. sp	Upper Paraná River floodplain, Paraná state, Brazil	Unspecified	Pavanelli et al. (2004), Takemoto et al. (2009), Luque et al. (2011)
*Contracaecum* sp. (larvae)	Upper Paraná River	Mesentery	Casali &Takemoto (2016)
	floodplain in Mato Grosso do Sul state (next to municipality of Porto Rico), Paraná state, Brazil; Riachuelo River Lagoon, Corrientes Province, Argentina		
		Intestinal mesenteries	Hamann (1999), Chemes & Takemoto (2011)
*Cucullanus* sp.	Upper Paraná River floodplain, Paraná state, Brazil	Unspecified	Pavanelli et al. (1997), Pavanelli et al. (2004), Takemoto et al. (2009), Luque et al. (2011)
*Eustrongylides ignotus* (Jägerskiöld, 1909)	Upper Paraná River floodplain, Brazil	Unspecified	Pavanelli et al. (2004), Takemoto et al. (2009), Luque et al. (2011)
*Eustrongylides* sp.	Riachuelo River Lagoon, Corrientes Province, Argentina	Visceral cavity (encysted larvae)	Chemes & Takemoto (2011)
Philometridae gen. sp.	Upper Paraná River floodplain, Paraná state, Brazil	Unspecified	Pavanelli et al. (2004), Takemoto et al. (2009), Luque et al. (2011)
*Procamallanus* sp.	Upper Paraná River floodplain, Paraná state, Brazil	Unspecified	Pavanelli et al. (1997)
*Procamallanus* (*Spirocamallanus*) *inopinatus* (Travassos, Artigas & Pereira, 1928)	Upper Paraná River floodplain; Riachuelo River Lagoon, Corrientes Province, Argentina; Upper Paraná River, Brazil	Unspecified; Pyloric caecum	Pavanelli et al. (2004), Takemoto et al. (2009), Luque et al. (2011), Chemes & Takemoto (2011), Casali & Takemoto (2016)
	floodplain in Mato Grosso do Sul state (next to municipality of Porto Rico), Paraná state, Brazil;		
*Procamallanus* (*Spirocamallanus*) *neocaballeroi* (Caballero-Deloya, 1977)	Upper Paraná River	Intestine	Casali & Takemoto (2016)
	floodplain in Mato Grosso do Sul state (next to municipality of Porto Rico), Paraná state, Brazil		

* Parasitological reports from the Paraná River basin address the species *Serrasalmus maculatus* and *Serrasalmus spilopleura* Kner, 1860, as they were all synonymized with *S. maculatus* (Jégu & dos Santos, 2001; Rossin et al., 2019). However, for the Northern Brazil basin, the identification of *S. spilopleura* is still valid, so records of *S. spilopleura* in the northern basins were not included in the review. Furthermore, it is noteworthy that the occurrence of *S. maculatus* is recorded for the Amazon and Paraguay-Paraná River basins (Froese & Pauly, 2020b), while *S. spilopleura* is restricted to the basins of the Northern region of Brazil (Jégu & dos Santos, 2001).

**Figure 4:**
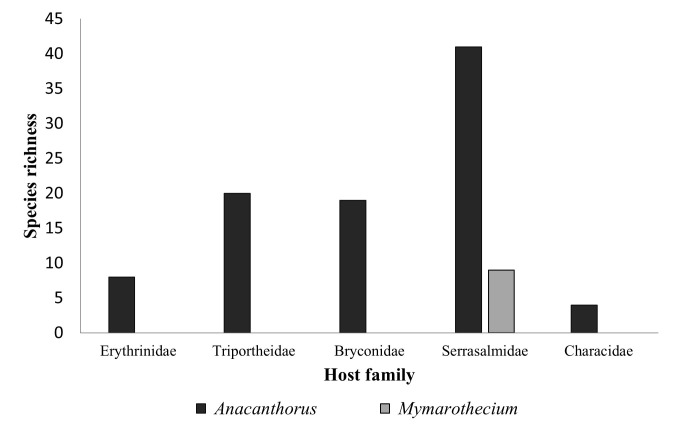
Monogeneans belonging to *Anacanthorus* and *Mymarothecium* (Dactylogyridae) reported from Neotropical characiform fishes.

Monogenean species belonging to *Anacanthorus* and *Mymarothecium* in Neotropical hosts comprise 101 species (Table 2 and Figure 4). The genus *Anacanthorus* includes ~92 valid species (Table 2 and Figure 4), which are gill parasites of characiform fishes of the Serrasalmidae (41 species), Triportheidae (20 species), Bryconidae (19 species), Erythrinidae (eight species), and Characidae (four species). Brazil harbours the greatest number of *Anacanthorus* taxa (84 species). Meanwhile, the genus *Mymarothecium* includes nine species, which are also parasites of characiform fishes of the family Serrasalmidae, specifically of the genera *Serrasalmus* (four species), and *Piaractus* (two species), from Brazil, Peru, and Bolivia (Table 2 and Figure 4)

**Table 2:** Checklist of valid species of monogeneans belonging to *Anacanthorus* and *Mymarothecium* (Dactylogyridae) reported in characiform fishes from Neotropical region.

	**Hosts**	**Host Family**	**Locality**	**Reference**
*Anacanthorus acrophallus* (Neto, Muriel-Cunha & Domingues, 2019)	*Hoplerythrinus unitaeniatus (Spix & Agassiz, 1829)*	Erythrinidae	Guamá River, Pará state, Brazil	Neto et al. (2019)
*Anacanthorus acuminatus* (Kritsky, Boeger & Van Every, 1992)	*Triportheus angulatus (Spix & Agassiz, 1829)*	Triportheidae	Furo do Catalão and Solimões River, Amazonas state, Brazil	Kritsky et al. (1992), Moreira et al. (2017)
	*Triportheus elongatus (Günther, 1864)*			
	*Triportheus albus (Cope, 1872)*			
*Anacanthorus adkruidenieri* (Monteiro, Cohen & Brasil-Sato, 2015)	*Salminus franciscanus (Lima & Britski, 2007)*	Bryconidae	São Francisco River, Minas Gerais state, Brazil	Monteiro et al. (2015)
*Anacanthorus alatus* (Kritsky, Boeger & Van Every, 1992)	*Triportheus albus*	Triportheidae	Furo do Catalão and Solimões River, Amazonas state, Brazil	Kritsky et al. (1992)
	*Triportheus elongatus*			
*Anacanthorus amazonicus* (Van Every & Kritsky, 1992)	*Serrasalmus rhombeus (Linnaeus, 1766); Serrasalmus sp.; Pristobrycon striolatus (Steindachner, 1908)*	Serrasalmidae	Pitinga, Uatumã and Negro Rivers, Amazonas state, Brazil	Van Every & Kritsky (1992)
	*Serrasalmus rhombeus*		San Martin, Beni and Ichilo Rivers, Bolivia	Córdova & Pariselle (2007)
	*Serrasalmus altispinis (Merckx, Jégu & Santos, 2000)*		Solimões and Purus Rivers, Amazonas state, Brazil.	Morey & Malta (2018)

*Anacanthorus anacanthorus* (Mizelle & Price, 1965)	*Pygocentrus nattereri Kner 1858 (=Serrasalmus nattereri Kner, 1858)*	Serrasalmidae	Amazonas River, Brazil;	Mizelle & Price (1965)
				Brito-Junior & Tavares-Dias (2018)
			Igarapé basin, Amapá state, Brazil	
*Anacanthorus andersoni* (Kritsky, Boeger & Van Every, 1992)	*Triportheus angulatus*	Triportheidae	São Jorge’s district, Manaus, Amazonas state, Brazil	Kritsky et al. (1992)
*Anacanthorus ataidei* (Neto, Muriel-Cunha & Domingues, 2019)	*Erythrinus erythrinus (Bloch & Schneider, 1801)*	Erythrinidae	Caeté and Moju Rivers, Pará state, Brazil	Neto et al. (2019)
*Anacanthorus beleophallus* (Kritsky, Boeger & Van Every, 1992)	*Serrasalmus eigenmanni Norman, 1929 (= Pristobrycon eigenmanni Norman, 1929)*	Serrasalmidae	Negro River, Amazonas state, Brazil	Kritsky et al. (1992)
*Anacanthorus bellus* (Kritsky, Boeger & Van Every, 1992)	*Triportheus albus*	Triportheidae	Furo do Catalão and Solimões River, Amazonas state, Brazil	Kritsky et al. (1992)
	*Triportheus elongatus*			
	*Triportheus sp.*			
*Anacanthorus bicuspidatus* (Cohen, Kohn & Boeger, 2012)	*Salminus brasiliensis (Cuvier, 1816)*	Bryconidae	Paraná River, Paraná state, Brazil;	Cohen et al. (2012)
	*Salminus hilarii (Valenciennes, 1850)*		Taquari River, São Paulo state, Brazil	Brandão et al. (2013)
*Anacanthorus brazilensis* (Mizelle & Price, 1965)	*Pygocentrus nattereri (= Serrasalmus nattereri)*	Serrasalmidae	Amazonas River, Brazil;	Mizelle & Price (1965)
				Brito-Junior & Tavares-Dias (2018)
			Igarapé basin, Amapá state, Brazil	
*Anacanthorus brevicirrus* (Monteiro, Kritsky & Brasil-Sato, 2010)	*Brycon orthotaenia (Günther, 1864)*	Bryconidae	São Francisco River, Minas Gerais state, Brazil	Monteiro et al. (2010)
*Anacanthorus brevis* (Mizelle & Kritsky, 1969)	*Brycon melanopteru (Cope, 1872)*	Bryconidae	Xeruiny River, Amazonas state, Brazil	Mizelle et al. (1969)
*Anacanthorus calophallus* (Kritsky, Boeger & Van Every, 1992)	*Triportheus elongatus*	Triportheidae	Solimões River, Amazonas state, Brazil; Manaus Fish Market, Amazonas state, Brazil	Kritsky et al.(1992)
*Anacanthorus camposbaci* Morey, Aliano & Grandez, 2019 (=*Anacanthorus camposbacae* Morey, Aliano & Grandez, 2019)	*Myloplus schomburgkii (Jardine, 1841)*	Serrasalmidae	Nanay River, Iquitos, Peru.	Morey et al. (2019)
*Anacanthorus carinatus* (Kritsky, Boeger & Van Every, 1992)	*Triportheus angulatus*	Triportheidae	São Jorge’s district, Manaus, Amazonas state, Brazil	Kritsky et al. (1992)
*Anacanthorus carmenrosae* (Morey, Aliano & Grandez, 2019)	*Myloplus schomburgkii*	Serrasalmidae	Nanay River, Iquitos, Peru.	Morey et al. (2019)
*Anacanthorus catoprioni* (Kritsky, Boeger & Van Every, 1992)	*Catoprion mento (Cuvier, 1819)*	Serrasalmidae	Uatumã River and Furo do Catalão, Amazonas state, Brazil	Kritsky et al. (1992)
*Anacanthorus chaunophallus* (Kritsky, Boeger & Van Every, 1992)	*Triportheus angulatus*	Triportheidae	Furo do Catalão River, Amazonas state, Brazil	Kritsky et al. (1992); Moreira et al. (2017)
*Anacanthorus chelophorus* (Kritsky, Boeger & Van Every, 1992)	*Triportheus angulatus*	Triportheidae	São Jorge’s district, Manaus, Amazonas state, Brazil; Furo do Catalão, Amazonas state, Brazil	Kritsky et al. (1992)
	*Triportheus sp.*			
*Anacanthorus cinctus* (Van Every & Kritsky, 1992)	*Pristobrycon striolatus*	Serrasalmidae	Uatumã River, Amazonas state, Brazil;	Van Every & Kritsky (1992); Morey & Malta (2018)
	*Serrasalmus altispinis*		Solimões and Purus Rivers, Amazonas state, Brazil	
*Anacanthorus circumspatulatus* (Neto, Muriel-Cunha & Domingues, 2019)	*Erythrinus erythrinus*	Erythrinidae	Caeté and Moju Rivers, Pará state, Brazil	Neto et al. (2019)
*Anacanthorus cladophallus* (Van Every & Kritsky, 1992)	*Serrasalmus spilopleura (Kner, 1860)*	Serrasalmidae	Solimões River, Manaus, Amazonas state, Brazil	Van Every & Kritsky (1992)
	*Serrasalmus altispinis*			
			Solimões and Purus River, Amazonas state, Brazil	Morey & Malta (2018)
*Anacanthorus cohenae* (Pereira, Mota, Paiva & Tavares, 2020)	*Markiana nigripinnis (Perugia, 1891)*	Characidae	Marginal lake to the road MS184, Corumbá, Mato Grosso do Sul state, Brazil	Pereira et al. (2020)
*Anacanthorus colombianus* (Kritsky & Thatcher, 1974)	*Salminus affinis (Steindachner, 1880)*	Bryconidae	Jamundi River, Colômbia	Kritsky & Thatcher (1974)
*Anacanthorus contortus* (Cohen, Kohn & Boeger, 2012)	*Salminus brasiliensis*	Bryconidae	Paraná River, Paraná state, Brazil;	Cohen et al. (2012)
	*Salminus hilarii*		Taquari River, São Paulo state, Brazil	Brandão et al. (2013)
*Anacanthorus cornutus* (Kritsky, Boeger & Van Every, 1992)	*Triportheus angulatus*	Triportheidae	São Jorge’s district, Manaus, Amazonas state, Brazil	Kritsky et al. (1992)
*Anacanthorus crytocaulus* (Van Every & Kritsky, 1992)	*Pristobrycon striolatus*	Serrasalmidae	Pitinga and Uatumã Rivers, Amazonas state, Brazil	Van Every & Kritsky (1992)
			Solimões and Purus Rivers, Amazonas state,Brazil	Morey & Malta (2018)
	*Serrasalmus altispinis*			
*Anacanthorus cururutuiensis* (Neto, Muriel-Cunha & Domingues, 2019)	*Hoplerythrinus unitaeniatus*	Erythrinidae	Caeté and Guamá Rivers, Pará state, Brazil	Neto et al. (2019)
*Anacanthorus cuticulovaginus* (Kritsky & Thatcher, 1974)	*Salminus qffinis*	Bryconidae	Jamundi River, Colômbia	Kritsky & Thatcher (1974)
*Anacanthorus daulometrus* (Cohen, Kohn & Boeger, 2012)	*Salminus brasiliensis*	Bryconidae	Paraná River, Paraná state, Brazil	Cohen et al. (2012)
	*Salminus franciscanus*		São Francisco River, Minas Gerais state, Brazil	Monteiro et al. (2015)
*Anacanthorus dipelecinus* (Kritsky, Boeger & Van Every, 1992)	*Roeboides myersii (Gill, 1870)*	Characidae	Solimões and Negro Rivers, Amazonas state, Brazil	Kritsky et al. (1992)
*Anacanthorus douradensis* (Cohen, Kohn & Boeger, 2012)	*Salminus brasiliensis*	Bryconidae	Paraná River, Paraná state, Brazil	Cohen et al. (2012)
*Anacanthorus elegans* (Kritsky, Thatcher & Kayton, 1979)	*Brycon melanopterus Cope, 1872*	Bryconidae	Janauacá Lake, Amazonas state, Brazil	Kritsky et al. (1979)
*Anacanthorus euryphallus* (Kritsky, Boeger & Van Every, 1992)	*Triportheus angulatus;* *T. elongatus;*	Triportheidae	Furo do Catalão, Amazonas state, Brazil; Manaus, Amazonas state, Brazil	Kritsky et al. (1992); Moreira et al. (2017)
	*T. albus*			
*Anacanthorus femoris* (Morey, Sol & Cachique, 2021)	*Brycon amazonicus (Spix e Agassiz, 1892)*	Bryconidae	River Tahuayo, Loreto state, Peru	Morey et al. (2021)
*Anacanthorus formosus* (Kritsky, Boeger & Van Every, 1992)	*Triportheus elongatus; Triportheus sp.*	Triportheidae	Furo do Catalão and Solimões River, Amazonas state, Brazil	Kritsky et al. (1992)
*Anacanthorus franciscanus* (Monteiro, Kritsky & Brasil-Sato, 2010)	*Brycon orthotaenia*	Bryconidae	São Francisco River, Minas Gerais state, Brazil	Monteiro et al. (2010)
*Anacanthorus furculus* (Kritsky, Boeger & Van Every, 1992)	*Triportheus elongatus*	Triportheidae	Solimões River, Amazonas state, Brazil	Kritsky et al. (1992)
			Manaus Fish Market, Amazonas state, Brazil	
*Anacanthorus glyptophallus* (Kritsky, Boeger & Van Every, 1992)	*Triportheus angulatus*	Triportheidae	São Jorge’s district, Manaus, Amazonas state, Brazil	Kritsky et al. (1992)
*Anacanthorus gravihamulatus* (Van Every & Kritsky, 1992)	*Serrasalmus rhombeus; Serrasalmus eigenmanni (=Pristobrycon eigenmanni); Serrasalmus sp.*	Serrasalmidae	Pitinga and Uatumã Rivers, Amazonas state, Brazil	Van Every & Kritsky (1992)
			Madre Dios River, Bolivia	Córdova & Pariselle (2007)
	*Serrasalmus rhombeus*			
	*Serrasalmus altispinis*		Matapi River, Amapá state, Brazil	Neves et al. (2020)
				Morey & Malta (2018)
			Solimões and Purus Rivers, Amazonas state, Brazil	
*Anacanthorus hoplophallus* (Kritsky, Boeger & Van Every, 1992)	*Myloplus rubripinnis (Müller & Troschel, 1844)*	Serrasalmidae	Uatumã River, Amazonas state, Brazil	Kritsky et al. (1992)
*Anacanthorus jegui* (Van Every & Kritsky, 1992)	*Serrasalmus spilopleura; Serrasalmus sp.; Serrasalmus eigenmanni (=Pristobrycon eigenmanni); Pristobrycon sp.*	Serrasalmidae	Solimões, Pitinga and Uatumã Rivers and Furo do Catalão, Amazonas state, Brazil;	Kritsky et al. (1992)
	*Serrasalmus rhombeus*		San Martin, Beni, Madre Dios and Ichilo Rivers,Bolivia	Córdova & Pariselle (2007)
				Hoshino & Tavares-Dias (2014) ; Neves et al. (2020) ;
			Igarapé basin, Amapá state, Brazil; Matapi River, Amapá state, Brazil	Morey & Malta, (2018)
	*Metynnis lippincottianus Cope, 1870*		Solimões and Purus Rivers, Amazonas state, Brazil	
	*Serrasalmus altispinis*			
*Anacanthorus kruidenieri* (Kritsky, Thatcher & Kayton, 1979)	*Brycon melanopterus*	Bryconidae	Janauacá Lake, Amazonas state, Brazil	Kritsky et al. (1979)
*Anacanthorus kukamensis* (Morey, Sol & Cachique, 2021)	*Brycon amazonicus*	Bryconidae	River Tahuayo, Loreto state, Peru	Morey et al. (2021)
*Anacanthorus lacinimentulatus* (Neto, Muriel-Cunha & Domingues, 2019)	*Hoplerythrinus unitaeniatus*	Erythrinidae	Guamá and Moju Rivers, Pará state, Brazil	Neto et al. (2019)
*Anacanthorus lasiophallus* (Van Every & Kritsky, 1992)	*Pristobrycon striolatus*	Serrasalmidae	Pitinga and Uatumã Rivers, Amazonas state, Brazil	Van Every & Kritsky (1992)
*Anacanthorus lepyrophallus* (Kritsky, Boeger & Van Every, 1992)	*Serrasalmus elongatus; Serrasalmus sp.*	Serrasalmidae	Negro and Solimões Rivers, Lago do Rei, Paraná, Ilha do Careiro and Furo do Catalão, Amazonas state, Brazil	Kritsky et al. (1992)
	*Serrasalmus altispinis*		Solimões and Purus Rivers, Amazonas state, Brazil	Morey & Malta (2018)
			Upper Paraná River floodplain, Paraná state, Brazil	
				Moreira et al. (2019)
			**Ilha Solteira reservoir, Upper Paraná River basin, São Paulo state, Brazil**	**Present study**
	*Serrasalmus maculatus*			
*Anacanthorus luquei* (Pereira, Mota, Paiva & Tavares, 2020)	*Markiana nigripinnis*	Characidae	Marginal lake to the road MS184, Corumbá, Mato Grosso do Sul state, Brazil	Pereira et al. (2020)
*Anacanthorus lygophallus* (Kritsky, Boeger & Van Every, 1992)	*Triportheus angulatus*	Triportheidae	Furo do Catalão, Amazonas state, Brazil	Kritsky et al. (1992), Moreira et al. (2017)
*Anacanthorus maltai* (Boeger & Kritsky, 1988)	*Pygocentrus nattereri (= Serrasalmus nattereri)*	Serrasalmidae	Mamoré River, Rondônia state, Brazil	Boeger & Kritsky (1988)
*Anacanthorus maratininguensis* (Neto, Muriel-Cunha & Domingues, 2019)	*Hoplerythrinus unitaeniatus*	Erythrinidae	Moju, Caeté and Guamá Rivers, Pará state, Brazil	Neto et al. (2019)
*Anacanthorus mastigophallus* (Kritsky, Boeger & Van Every, 1992)	*Serrasalmus eigenmanni (=Pristobrycon eigenmanni)*	Serrasalmidae	Uatumã and Pitinga Rivers, Amazonas state, Brazil	Kritsky et al. (1992)
*Anacanthorus mesocondylus* (Van Every & Kritsky, 1992)	*Serrasalmus elongatus; Serrasalmus rhombeus; Serrasalmus spilopleura; Serrasalmus sp; Serrasalmus eigenmanni (=Pristobrycon eigenmanni); Pristobrycon sp.*	Serrasalmidae	Solimões, Negro, Uatumã and Pitinga Rivers, Amazonas state, Brazil	Van Every & Kritsky (1992)
	*Serrasalmus altispinis*		Solimões and Purus Rivers, Amazonas state, Brazil	Morey & Malta (2018)

*Anacanthorus myleusi* (Moreira, Carneiro, Ruz & Luque, 2019)	*Myloplus schomburgkii*	Serrasalmidae	Xingu River, Pará state, Brazil.	Moreira et al. (2019)
*Anacanthorus nanus* (Kritsky, Boeger & Van Every, 1992)	*Triportheus angulatus*	Triportheidae	Bairro de São Jorge, Manaus, Amazonas state, Brazil	Kritsky et al. (1992)
*Anacanthorus neotropicalis* (Mizelle & Price, 1965)	*Pygocentrus nattereri (= Serrasalmus nattereri)*	Serrasalmidae	Amazonas River, Brazil	Mizelle & Price (1965)
*Anacanthorus palamophallus* (Kritsky, Boeger & Van Every, 1992)	*Serrasalmus eigenmanni (=Pristobrycon eigenmanni) Salminus franciscanus*	Serrasalmidae	Uatumã River, Amazonas state, Brazil	Kritsky et al. (1992)
*Anacanthorus paradouradensis* (Monteiro, Cohen & Brasil-Sato, 2015)	*Salminus franciscanus*	Bryconidae	São Francisco River, near to Três Marias reservoir, Minas Gerais state, Brazil	Monteiro et al. (2015)
*Anacanthorus parakruidenieri* (Cohen, Kohn & Boeger, 2012)	*Salminus brasiliensis*	Bryconidae	Paraná River, Paraná state, Brazil	Cohen et al. (2012)
*Anacanthorus paraspathulatus* (Kritsky, Boeger & Van Every, 1992)	*Mylossoma duriventris (Cuvier, 1817)*	Serrasalmidae	Solimões River, Amazonas state, Brazil	Kritsky et al. (1992);
			Lake Coari, Amazonas state, Brazil	Silva & Tavares-Dias (2012)
	*Mylossoma aureum (Spix & Agassiz, 1829)*		Guandu River, Rio de Janeiro state, Brazil	Azevedo et al. (2011)
*Anacanthorus paraxaniophallus* (Moreira, Carneiro, Ruz & Luque, 2019)	*Serrasalmus maculatus; Serrasalmus marginatus*	Serrasalmidae	Miranda River, Pantanal, Mato Grosso do Sul state, Brazil	Moreira et al. (2019)
*Anacanthorus pedanophallus* (Kritsky, Boeger & Van Every, 1992)	*Myloplus rubripinnis Müller & Troschel, 1844 (=Myleus rubripinnis)*	Serrasalmidae	Uatumã River, Amazonas state, Brazil	Kritsky et al. (1992)
*Anacanthorus pelorophallus* (Kritsky, Boeger & Van Every, 1992)	*Triportheus elongatus*	Triportheidae		Kritsky et al. (1992)
			Solimões River, Amazonas state, Brazil	
			Manaus Fish Market, Manaus, Amazonas state, Brazil	
*Anacanthorus penilabiatus* (Boeger, Husak & Martins, 1995)	*Piaractus mesopotamicus*	Serrasalmidae	Aquaculture Center, UNESP, São Paulo state, Brazil	Boeger et al. (1995)
			“Departamento Nacional de Obras Contra as Secas, DNOCS”, Ceará state, Brazil	
	*Piaractus mesopotamicus*			Pamplona-Basilio et al. (2001); Cohen & Kohn (2009)
	*Piaractus brachypomus*			Leão et al. (2017)
	
	*Colossoma macropomum*			
			Itaipu reservoir, Paraná River, Paraná state, Brazil	
	
	*Piaractus mesopotamicus*			
*Anacanthorus periphallus* (Kritsky, Boeger & Van Every, 1992)	*Serrasalmus sp.*	Serrasalmidae	Furo do Catalão and Solimões River, Amazonas state, Brazil	Kritsky et al. (1992)
			Solimões and Purus Rivers, Amazonas state, Brazil	Morey & Malta (2018)
	*Serrasalmus altispinis*			
*Anacanthorus pithophallus* (Kritsky, Boeger & Van Every, 1992)	*Triportheus angulatus*	Triportheidae	São Jorge' district, Manaus,	Kritsky et al. (1992), Moreira et al. (2017)
			Amazonas state, Brazil; Catalão floodplain lake Manaus, Amazonas state, Brazil	
*Anacanthorus prodigiosus* (Van Every & Kritsky, 1992)	*Serrasalmus elongatus; Serrasalmus rhombeus; Serrasalmus sp.*	Serrasalmidae	Negro, Solimões, Uamatã, and Pitinga Rivers, Amazonas, Brazil	Kritsky et al. (1992)
	*Serrasalmus altispinis*		Solimões River, Amazonas state, Brazil	Morey & Malta, (2018)
*Anacanthorus quinqueramus* (Kritsky, Boeger & Van Every, 1992)	*Triportheus albus; Triportheus elongatus; Triportheus sp.*	Serrasalmidae	Furo do Catalão and Solimões River, Amazonas state, Brazil	Kritsky et al. (1992)
*Anacanthorus ramosissimus* (Van Every & Kritsky, 1992)	*Serrasalmus elongatus*	Serrasalmidae	Solimões River, Amazonas state, Brazil	Van Every & Kritsky (1992)
*Anacanthorus ramulosus* (Kritsky, Boeger & Van Every, 1992)	*Triportheus albus; Triportheus elongatus*	Triportheidae	Furo do Catalão, Amazonas state, Kritsky et al. (1992) Brazil	Kritsky et al. (1992)
*Anacanthorus rarus* (Morey, Sol & Cachique, 2021)	*Brycon amazonicus*	Bryconidae	River Tahuayo, Loreto state, Peru	Morey et al. (2021)
*Anacanthorus reginae* (Boeger & Kritsky, 1988)	*Pygocentrus nattereri (= Serrasalmus nattereri)*	Serrasalmidae	Solimões River, Amazonas state, Brazil	Boeger & Kritsky (1988)
				Morais & Malta (2015)
				Iannacone & Luque (1993)
			Amazonas River, Peru	
*Anacanthorus rondonensis* (Boeger & Kritsky, 1988)	*Pygocentrus nattereri (= Serrasalmus nattereri)*	Serrasalmidae	Mamoré River, Rondônia state, Brazil;	Boeger & Kritsky (1988)
	*Serrasalmus rhombeus*			Córdova & Pariselle (2007)
			Madre de Dios River, Bolívia	
*Anacanthorus sabaloi* (Morey, Sol & Cachique, 2021)	*Brycon amazonicus*	Bryconidae	River Tahuayo, Loreto state, Peru	Morey et al. (2021)
*Anacanthorus scapanus* (Van Every & Kritsky, 1992)	*Serrasalmus spilopleura*	Serrasalmidae	Solimões River, Amazonas state, Brazil	Van Every & Kritsky (1992)
*Anacanthorus scholzi* (Pereira, Mota, Paiva & Tavares, 2020 )	*Markiana nigripinnis*	Characidae	Marginal lake to the road MS184, Corumbá, Mato Grosso do Sul state, Brazil	Pereira et al. (2020)
*Anacanthorus sciponophallus* (Van Every & Kritsky, 1992)	*Serrasalmus elongatus; Serrasalmus rhombeus; Serrasalmus spilopleura; Serrasalmus sp.*	Serrasalmidae	Solimões, Negro, Pitinga, Uatumã Rivers, and Ilha do Careiro, Amazonas state, Brazil	Van Every & Kritsky (1992)
	*Serrasalmus rhombeus*			
			San Martin, Beni, Madre Dios and Ichilo Rivers, Bolivia	Córdova & Pariselle (2007)
			Solimões and Purus Rivers, Amazonas state, Brazil	Morey & Malta (2018)
	
	*Serrasalmus altispinis*			

			Batalha and Peixe Rivers, Upper Paraná River, São Paulo state, Brazil	Dias et al. (2017)
	
	*Serrasalmus maculatus*			

*Anacanthorus serrasalmi* Van (Every & Kritsky, 1992)	*Serrasalmus rhombeus; Serrasalmus elongatus; Serrasalmus sp.; Pristobrycon sp.*	Serrasalmidae	Pitinga, Uatumã, Negro, and Solimões Rivers, Amazonas state, Brazil;	Van Every & Kritsky (1992)
	*Serrasalmus altispinis*		Solimões and Purus Rivers, Amazonas state, Brazil	Morey & Malta (2018)
*Anacanthorus siphonocommus* (Neto, Muriel-Cunha & Domingues, 2019)	*Hoplerythrinus unitaeniatus*	Erythrinidae	Caeté and Guamá Rivers, Pará state, Brazil	Neto et al., (2019)
*Anacanthorus spathulatus* (Kritsky, Thatcher & Kayton, 1979)	*Piaractus brachypomus (=Colossoma bidens); Colossoma macropomum*	Serrasalmidae	Janauacá Lake, Amazonas state, Brazil	Kritsky et al. (1979)
	*Colossoma macropomum*			Fischer et al. (2003)
			Solimões and Amazon Rivers, Pará, Brazil	
	*Colossoma macropomum*		“Experimental Papelón, del Instituto Nacional de Investigaciones Agropecuarias”, Portuguesa state, Venezuela	
	*Híbrido (C. macropomum x Piaractus brachypomus)*		Delta Amacuro Experimental Station of the National Institute of Agricultural Research (INIA), Venezuela	
				Aragort et al. (2002)

				Centeno et al. (2004)
*Anacanthorus spinatus* (Kritsky, Boeger & Van Every, 1992)	*Myloplus rubripinnis (=Myleus rubripinnis)*	Serrasalmidae	Uatumã River, Amazonas state, Brazil	Kritsky et al. (1992)
*Anacanthorus spiralocirrus* (Kritsky, Thatcher & Kayton, 1979)	*Brycon melanopterus*	Bryconidae	Janauacá Lake, Amazonas state, Brazil	Kritsky et al. (1979)
			Amazonas state, Brazil	
	*Brycon amazonicus*		River Tahuayo, Loreto state, Peru	Andrade & Malta (2006);
				Morey et al. (2021)

*Anacanthorus stachophallus* (Kritsky, Boeger & Van Every, 1992)	*Pygocentrus nattereri (= Serrasalmus nattereri)*	Serrasalmidae	Solimões River and Furo do Catalão, Amazonas state, Brazil;	Kritsky et al. (1992)
			Solimões River, Amazonas state, Brazil	
				Morais & Malta (2015)
			Amazonas River, Peru	Iannacone & Luque (1993)
*Anacanthorus stagmophallus* (Kritsky, Boeger & Van Every, 1992)	*Myloplus rubripinnis (=Myleus rubripinnis)*	Serrasalmidae	Uatumã River, Amazonas state, Brazil	Kritsky et al. (1992)
*Anacanthorus strongylophallus* (Kritsky, Boeger & Van Every, 1992)	*Triportheus elongatus*	Triportheidae	Solimões River, Amazonas state, Brazil	Kritsky et al. (1992)
*Anacanthorus thatcheri* (Boeger & Kritsky, 1988)	*Pygocentrus nattereri (= Serrasalmus nattereri)*	Serrasalmidae	Solimões River, Amazonas state, Brazil	Boeger & Kritsky (1988); Morais & Malta (2015)
			Amazonas River, Peru	Iannacone & Luque (1993)
*Anacanthorus toledoensis* (Leão, São Clemente & Cohen, 2015)	*Piaractus mesopotamicus*	Serrasalmidae	Paraná River, Paraná state, Brazil	Leão et al., (2015)
*Anacanthorus tricornis* (Kritsky, Boeger & Van Every, 1992)	*Triportheus elongatus; T. angulatus*	Triportheidae	Solimões River and Manaus Fish Market, Amazonas state, Brazil;	Kritsky et al. (1992)
			São Jorge’s district, Manaus;	
			and Furo do Catalão, Amazonas state, Brazil	
*Anacanthorus xaniophallus* (Kritsky, Boeger & Van Every, 1992)	*Serrasalmus eigenmanni (=Pristobrycon eigenmanni); Pristobrycon sp.*	Serrasalmidae	Uatumã River, Amazonas state, Brazil	Kritsky et al. (1992)
			Negro River, Amazonas state Brazil	
*Mymarothecium boegeri* (Cohen & Kohn, 2005)	*Colossoma macropomum*	Serrasalmidae	Aquarium from “Centro de Pesquisas em	Cohen & Kohn (2005); Cohen & Kohn (2009)
			Aquicultura Rodolfo von Ihering, DNOCS”, Ceará state, Brazil	
				Dias et al. (2015)
	*Híbrido (Colossoma macropomum x Piaractus brachypomus)*			
			Matapi, Amapá state, Brazil	
*Mymarothecium dactylotum* (Kritsky, Walter, Boeger & Jegu, 1996)	*Serrasalmus rhombeus*	Serrasalmidae	Pitinga, Uatumã, Negro and Jatapú Rivers, Cachoeira das Garças, Furo do Catalão, Amazonas state, Brazil	Kritsky et al. (1996)
	*Pristobrycon sp.*			
	*Serrasalmus sp.*			
*Mymarothecium galeolum* (Kritsky, Walter, Boeger & Jegu, 1996)	*Serrasalmus eigenmanni (=Pristobrycon eigenmanni); Pristobrycon sp.; Serrasalmus gouldingi (Fink & Machado-Allison 1992); Serrasalmus rhombeus*	Serrasalmidae	Uatumã, Jatapú, Pitinga and Negro Rivers, Cachoeira das Garças, Furo do Catalão Amazonas state, Brazil	Kritsky et al. (1996)
	*Serrasalmus rhombeus*			

			San Martín, Madre de Dios and Ichilo Rivers, Bolívia	
				Córdova & Pariselle (2007)
*Mymarothecium ianwhittingtoni* (Leão, São Clemente & Cohen, 2015)	*Piaractus mesopotamicus*	Serrasalmidae	Paraná River, Toledo, Paraná state, Brazil	Leão et al. (2015)
			Itaipu reservoir, Paraná River, Paraná state, Brazil	Leão et al. (2017)
*Mymarothecium iiapense* Morey, Aliano & Grandez, 2019 (=*Mymarothecium iiapensis* Morey, Aliano e Grandez, 2019 )	*Colossoma macropomum*	Serrasalmidae	Fishpond from the “Centro de Investigações Fernando Alcántara Bocanegra (CIFAB), Instituto de Investigações de la Amazônia Peruana (IIAP)”, Iquitos, Peru	Morey et al. (2019)

*Mymarothecium perplanum* (Kritsky, Walter, Boeger & Jegu, 1996)	*Serrasalmus spilopleura*	Serrasalmidae	Uatumã and Solimões Rivers, Amazonas state, Brazil	Kritsky et al. (1996)
*Mymarothecium tantaliani* (Cayulla-Quispe, Mondragón-Martínez, Rojas-De-Los-Santos, Garcia-Candela, Babilonia-Medina & Martínez-Rojas, 2020)	*Colossoma macropomum*	Serrasalmidae	Puerto Maldonado, Madre de Dios River, Peru	Cayulla-Quispe et al. (2021)
*Mymarothecium viatorum* (Boeger, Piasecki & Sobecka, 2002)	*Piaractus brachypomus (Cuvier, 1818); Piaractus mesopotamicus (Holmberg, 1887)*	Serrasalmidae	Aquarium of the “Centro de Pesquisas em Aquicultura Rodolfo von Ihering, DNOCS”, Ceará state, Brazil;	Cohen & Kohn (2005)
	*Híbrido (Colossoma macropomum x Piaractus mesopotamicus)*		Piscicultures from municipality of Macapá, Amapá state, Brazil.	
	*Piaractus mesopotamicus*			Silva et al. (2013)
		
	*Híbrido “patinga” (P. mesopotamicus x Piaractus brachypomus)*		Piscicultures from municipality of Estrela d’Oeste, São Paulo state, Brazil	
				Franceschini et al. (2013)
	*Piaractus mesopotamicus*		Itaipu reservoir, Paraná River, Paraná state, Brazil	
				Leão et al. (2017)
*Mymarothecium whittingtoni* (Kritsky, Walter, Boeger & Jegu, 1996)	*Serrasalmus sp.; Serrasalmus rhombeus; Serrasalmus spilopleura;*	Serrasalmidae	Solimões River, Furo do Catalão, Ilha do Careiro, Amazonas state, Brazil	Kritsky et al. (1996)
*Mymarothecium* sp.	*Serrasalmus maculatus*	Serrasalmidae	Ilha Solteira reservoir, Upper Paraná River basin. São Paulo state, Brazil	**Present study**

Stomach content analysis resulted in the identification of ten food items, which mostly included fish fragments (81.7%) but also included terrestrial plants and decapods (*Macrobrachium* sp.) (Table 3). *Serrasalmus maculatus* showed piscivorous food habits, due to the predominant consumption of fish fragments (81.7%).

**Table 3:** Dietary components of piranha *Serrasalmus maculatus* specimens collected from the Ilha Solteira hydroelectric reservoir, Upper Paraná River basin, São Paulo state, Brazil.

**Food items**	% Volume
Fish fragments	81.7
Terrestrial plants	7.7
Decapoda (*Macrobrachium* sp.)	4.6
Gastropoda	1.8
Odonata	0.9
Other aquatic invertebrates	0.9
Remains of terrestrial insects	0.9
Aquatic plants	0.6
Seed	0.5
Detritus	0.4

## Discussion

This is the first study to report the parasitic fauna of *S. maculatu*s from the northwest region of the Upper Paraná River basin, São Paulo, Brazil. In addition, represents the first report of monogeneans belonging to *Mymarothecium* in this host species and first report of *Anacanthorus lepyrophallus* in the Ilha Solteira Reservoir. For monogeneans that parasitise fish gills, the phylogenetic relationships and evolutionary history between host orders are important factors for host-parasite interaction and distribution (Braga et al., 2014).

Previous studies have demonstrated that most monogeneans prefer to parasitise specific host lineages (Graça et al., 2018; Moreira et al., 2019) (e.g., *Mymarothecium* taxa parasitise members of the Serrasalmidae) (Braga et al., 2015). However, in some cases, members of other monogenean families have been reported to colonize phylogenetically distant hosts. In both cases, host-parasite relationships result from a combination of factors, including cospeciation, host-switching, and ecological fitting (Janzen, 1985; Brooks et al., 2006; Braga et al., 2014, 2015). Considering the monophyly of the Characiformes and the diversification of the group only in the continental neotropics, the phylogenetic contiguity between the order’s families may indicate the sharing of a range of intrinsic resources (Braga et al., 2015). *Anacanthorus* spp. are widely distributed in hosts of the five families of the order Characiformes (Figure 4). The sharing of resources (e.g., phylogenetic conservatism and phenotypic flexibility) may have favoured its occurrence within individuals of the same order and family (see Braga et al., 2014, 2015 and cited references).

The predominance of monogeneans in *S. maculatus* in Neotropical region could be associated with both the parasites’ monoxenous biology and host species’ gregarious habit (Sazima & Machado, 1990; Strona, 2015). Indeed, the proximity of fish in shoals can facilitate monogenean transmission, which occurs through simple contact between hosts (Thatcher, 2006). Furthermore, gregarious behaviour also allows free-native larval forms (oncomiracidia) to locate hosts more easily (Thatcher, 2006), which would justify the results observed in the present study.

The low parasite richness and absence of endoparasites observed in the present study may be related to host behaviour and/or foraging. Several studies have reported that heteroxenous parasites are transmitted *via* food web interactions and that intermediate hosts are nearly always dietary components of the parasites’ definitive hosts (Luque & Poulin, 2008; Lima et al., 2016). Therefore, host diet is considered an important factor in host-parasite interactions, and hosts with more diverse diets tend to be more susceptible to endoparasite infections and, thus, usually harbour greater parasite richness (Lima et al., 2016).

The dietary components of *S. maculatus* identified in the present study were like the findings of previous studies in the Upper Paraná floodplain, including the Ibicuí River, Rio Grande do Sul state, and a lower stretch of the Sorocaba River basin, São Paulo state, Brazil (Agostinho & Marques, 2001; Agostinho et al., 2003; Behr & Signor, 2008; Villares et al., 2008). *Serrasalmus maculatus* is piscivorous, preferentially ingesting fish fragments (instead of ingesting the host's entire body), and its feeding behaviour includes the mutilation of prey scales, fins, and muscle tissue, which we infer can hinder the ingestion of endoparasites (Sazima & Pombal-Jr, 1988; Sazima & Machado, 1990; Casali & Takemoto, 2016). In the present study, the dietary components of *S. maculatus* were fish fragments, terrestrial plants, and decapods (*Macrobrachium* sp.). However, even though *Macrobrachium* sp. is one of the most common of *S. maculatus*’ prey items, this genus of shrimp is native from Amazon basin (Collart & Moreira, 1993), and was introduced in Paraná River basin (Bialetzki et al., 1997). When a species is introduced to a new area, it may lose part of its natural parasite fauna (i.e., Enemy Release Hypothesis - Keane & Crawley, 2002; Tourchin et al., 2002; Mitchell & Power, 2003; Torchin et al., 2003) and, thereby, break the natural network of complex interactions between intermediate and definitive hosts, which alters the infection dynamics and enables the loss of parasite taxa (Madi & Ueta, 2009).

Several authors have reported rich endoparasite fauna for *S. maculatus* in the Upper Paraná River floodplain, whereas endoparasites were completely absent in the present study, and the richness of ectoparasites was low (Pavanelli et al., 1997; Pavanelli et al., 2004; Takemoto et al., 2009; Casali & Takemoto, 2016 ‒ see Table 1). It is possible that the dynamics of parasitic infections are negatively affected by abiotic and biotic homogenisation in artificial habitats (Agostinho et al., 2007), such as hydroelectric reservoirs, especially for endoparasites with heteroxenous life cycles.

Floodplains are highly dynamic and complex systems because they include a wide variety of aquatic habitats (e.g., rivers, lakes, and canals) (Junk, 1980; Power et al., 1995), when compared to artificial reservoirs, since the hydrodynamics and biotic communities of such last environments are altered during the damming process. The conversion of lotic to lentic environments involves a series of negative biotic and abiotic impacts, including changes in flow and channel granulometry, increases in fish mortality, increased predation rates, simplification of trophic chains, interruption of fish migration, eutrophication, deterioration of water quality, reduction of benthic community stability, colonisation by macrophytes, invasion by non-native species, and simplification of habitats (Agostinho et al., 1992; 2008). Furthermore, these changes can ultimately reduce the abundance and richness of local biota, disrupt the dynamics of host-parasite relationships, and, consequently, alter the structure of parasitic communities (Morley, 2007), and these seem to be the drivers involved here regarding the low parasite richness observed for *S. maculatus.*

In summary, the richness of the component parasite community of *S. maculatus* in the Ilha Solteira hydroelectric reservoir in Brazil was low, in contrast to what has been previously reported in other water environments (Pavanelli et al., 1997, 2004; Takemoto et al., 2009; Casali & Takemoto, 2016). These findings provide insight into the relationships between *S. maculatus* diet, social behaviour, and parasite fauna and the distribution and infection patterns of the observed parasite taxa. The present study also illustrates the possible effects of habitat homogenisation on parasite infection dynamics in artificial reservoirs. However, additional multidisciplinary research is needed to elucidate the effects of biotic and abiotic factors on the structure and dynamics of component communities of fish parasites in natural and artificial habitats in the neotropics.
